# Neuroevolution Guided Hybrid Spiking Neural Network Training

**DOI:** 10.3389/fnins.2022.838523

**Published:** 2022-04-25

**Authors:** Sen Lu, Abhronil Sengupta

**Affiliations:** School of Electrical Engineering and Computer Science, The Pennsylvania State University, University Park, PA, United States

**Keywords:** Spiking Neural Networks, neuroevolution, adversarial attack, neuromorphic computing, hybrid training

## Abstract

Neuromorphic computing algorithms based on Spiking Neural Networks (SNNs) are evolving to be a disruptive technology driving machine learning research. The overarching goal of this work is to develop a structured algorithmic framework for SNN training that optimizes unique SNN-specific properties like neuron spiking threshold using neuroevolution as a feedback strategy. We provide extensive results for this hybrid bio-inspired training strategy and show that such a feedback-based learning approach leads to explainable neuromorphic systems that adapt to the specific underlying application. Our analysis reveals 53.8, 28.8, and 28.2% latency improvement for the neuroevolution-based SNN training strategy on CIFAR-10, CIFAR-100, and ImageNet datasets, respectively in contrast to state-of-the-art conversion based approaches. The proposed algorithm can be easily extended to other application domains like image classification in presence of adversarial attacks where 43.2 and 27.9% latency improvements were observed on CIFAR-10 and CIFAR-100 datasets, respectively.

## 1. Introduction

Spiking Neural Network (SNN) based next-generation brain-inspired computational paradigms are emerging to be a disruptive technology driving machine learning research due to its unique temporal, event-driven behavior. SNN computing models are driven by the fact that biological neurons process information temporally and the computation is triggered by sparse events or spikes transmitted from fan-in neurons. Recent work has demonstrated that event-driven SNNs can result in a significant reduction of power consumption and communication overhead in hardware implementations of Artificial Intelligence (AI) platforms by *exploiting “dynamic” sparsity in neural activations* (Merolla et al., 2014; Davies et al., 2018). In addition to event-driven computing in the network itself, such a computing framework is an ideal fit for the emerging market of low-power, low-latency event-driven sensors (Gallego et al., 2019) that capture spatio-temporal information in the spiking domain. Such an end-to-end pipeline across the stack from sensors to the hardware and computational primitives enables us to truly leverage advantages from event-driven computation and communication.

While the true potential of SNNs is expected to be demonstrated on spatio-temporal application drivers triggered by sparse events (Gallego et al., 2019; Mahapatra et al., 2020) by leveraging its temporal processing capability (Shrestha and Orchard, 2018; Neftci et al., 2019), significant research has been also performed to establish its near-term efficacy on standard static recognition tasks (Rueckauer et al., 2017; Sengupta et al., 2019; Wu et al., 2019; Lu and Sengupta, 2020; Rathi and Roy, 2020; Rathi et al., 2020; Deng and Gu, 2021), routinely performed by conventional deep learning methods [referred to as Analog Neural Networks (ANNs) (Diehl et al., 2015), hereafter]. The vast majority of works in this domain have focused on rate encoding frameworks (Diehl et al., 2015) where the operation of the ANN is distributed as binary information over time in the SNN, resulting in a significant reduction of peak power consumption (Singh et al., 2020). To achieve supervised SNN training, two competing approaches are usually adopted:

**(i) ANN-SNN conversion:** In this scenario, an ANN is trained with specific constraints (Diehl et al., 2015; Rueckauer et al., 2017; Sengupta et al., 2019; Lu and Sengupta, 2020) and subsequently converted to an SNN for event-driven inference on neuromorphic hardware. The conversion process is enabled by the equivalence of Rectified Linear Unit (ReLU) functionality of ANN neurons to the operation of an Integrate-Fire (IF) spiking neuron. The method takes advantage of standard ANN backpropagation techniques like stochastic gradient descent but is limited by the baseline ANN accuracy. Recent works have been directed at minimizing the accuracy loss during the conversion process (Lu and Sengupta, 2020; Deng and Gu, 2021) and have reported competitive accuracies in large-scale machine learning tasks.

**(ii) Direct SNN training:** Direct SNN training by adopting backpropagation through time (BPTT) has also proven successful recently, albeit in simpler image classification tasks. Gradient descent is usually performed in SNNs by approximating the spiking neuron operation by surrogate gradients to avoid the discontinuity in the neuron transfer function due to discrete spiking behavior (Shrestha and Orchard, 2018; Neftci et al., 2019). While SNN training from scratch would probably benefit from temporal processing in neuromorphic chips, current near-term GPU-enabled training suffers from limited scalability due to exploding memory requirements for BPTT.

Relative advantages and disadvantages of the two approaches are still being explored. Initial work has suggested that direct SNN training from scratch (Lee et al., 2020) or a hybrid method of fine-tuning an ANN-SNN converted network for a few epochs through BPTT (Rathi et al., 2020) can significantly reduce the SNN inference latency. However, recent approaches have shown that significant latency reduction can be also achieved through simple design-time and run-time optimizations in the ANN-SNN conversion process as well (Lu and Sengupta, 2020). This is also intuitive since the application drivers for image classification tasks are static and do not involve temporal information. Also, gradient descent is utilized to minimize the classification error in the rate encoding framework for both scenarios and not the inference latency. The ANN-SNN conversion process essentially abstracts the SNN operation in a time-averaged fashion during the training process without exploiting precise timing information for gradient descent.

This article is an attempt to develop a structured algorithmic framework for the ANN-SNN conversion process. The key parameter driving the event-driven temporal behavior of neurons in the SNN is the neuron threshold. A higher neuron threshold is useful for distinguishability of temporal evidence integration (Sengupta et al., 2019) and therefore translates to higher accuracy. A higher threshold also causes the neurons to spike less frequently thereby increasing the spiking sparsity at the cost of increased latency. Inference latency (impacting delay) and sparsity of the spike train (impacting power) are key metrics governing the energy efficiency (delay × power) of SNNs implemented on neuromorphic hardware. Hence, we can abstract the SNN network performance (accuracy/latency/power/energy) to be a function of the neuron thresholds in each layer of the network. It is worth mentioning here that all neurons in a particular layer have the same threshold to ensure consistent rate encoded information in each layer. Previous works have mainly optimized neuron thresholds to maximize accuracy (Sengupta et al., 2019) or adopt a sub-optimal heuristic choice for all thresholds in the network to reduce inference latency with minimal accuracy drop (Lu and Sengupta, 2020). However, different layers' thresholds of a network may have varying non-linear impact on the SNN efficiency metric and a holistic singular choice for the entire network may not be optimal. Further, the thresholds may also need to be re-tuned for different efficiency metrics of choice. Driven by this observation, we propose a hybrid training framework where a converted SNN is optimized in tandem with a neuroevolutionary algorithm. Once an ANN with appropriate constraints for conversion has been trained, we optimize the layerwise threshold using a neuroevolutionary algorithm. Neuroevolution optimized neural networks is a growing topic of interest (Stanley et al., 2019) guided by the notion that biological brains are an outcome of natural evolution. It is worth mentioning here that our proposal is not specific to the optimizer. We chose evolutionary algorithms due to their simple gradient-free operation, parallelizability, and ability to outperform reinforcement learning algorithms at scale (Such et al., 2017; Stanley et al., 2019). The neuroevolution process considers a space of possible candidate solutions (defined by a set of layerwise neuron thresholds) and evaluates a cost function (latency, accuracy, among others) by evaluating the candidate SNN on a subset of the training set through the evolution generations. The additional computing overhead due to the hybrid approach is therefore driven by relatively cheaper SNN inference runs. The main contributions of the article can be summarized as:

**(i)** We present a simple automated framework to optimize an SNN by a hybrid training process that does not suffer from computationally expensive operations like BPTT.

**(ii)** We evaluate our approach with regards to SNN inference latency improvements on static image classification tasks and adversarial attack scenarios (CIFAR-10, CIFAR-100, and ImageNet datasets). The framework can be easily extended to involve hardware aware constraints as well like peak power or energy consumption in specific layers.

**(iii)** We present design insights to interpret the optimized SNN thresholds. For image classification and adversarial attack scenarios, we obtain an interpretable understanding of the need for layerwise SNN optimization.

## 2. Related Works

**Hybrid SNN training:** Prior work has considered hybrid SNN training approaches (Lee et al., 2018, 2020; Rathi and Roy, 2020; Rathi et al., 2020). Relevant to our approach, Rathi and Roy (2020) considered training an ANN-SNN converted spiking network through BPTT for a few epochs to improve on inference latency. However, during the second stage of BPTT training, gradient descent was performed not only on the weights, but also on the neuron thresholds and additional leak parameters that were introduced in this second training stage. The requirement of joint optimization of weights and thresholds may not be necessary since the ratio of the two governs the spiking neuron behavior (Sengupta et al., 2019). Further, optimization of additional leak parameters adds to the computational burden of SNN BPTT. It is also unclear whether the superior SNN performance is attributed primarily to a fine-tuned optimized threshold or whether time-based information in training also plays a role.

In contrast, our algorithm adopts a simplistic approach of fine-tuning only the SNN thresholds to optimize the metric of choice using neuroevolutionary algorithms. Neuroevolutionary algorithms are easily parallelizable and the search parameter space in our scenario is bounded by the number of network layers and hence is not computationally expensive. The search process also involves evaluation of the cost function which is equivalent to the relatively cheaper SNN inference simulations and does not suffer from the explosive computational requirements of BPTT. The work also aims to serve as a benchmark for static image classification tasks to address the question of whether BPTT training from scratch or fine-tuning adds significantly to the training process. We provide results to substantiate that conversion techniques might produce competitive SNNs in application drivers not exploiting temporal information.

**Neuroevolution in SNN training:** Evolutionary algorithms have been used for training SNNs (Schuman et al., 2016, 2020; Elbrecht and Schuman, 2020) where the computational unit (neuron/synapse) parameters and network architectures have been optimized. A variety of techniques like EONS (Schuman et al., 2020) and HyperNEAT (Elbrecht and Schuman, 2020) algorithms have been used to train the networks from scratch. However, the techniques have been primarily evaluated on simple machine learning tasks. Hence, the scalability of the approaches remains unclear. Our work considers a hybridized approach where a supervised conventional SNN is optimized with a neuroevolutionary algorithm depending on the cost function of choice (accuracy, latency, among others), thereby leveraging the scalability of gradient descent approaches.

**Significance driven layerwise optimizations:** There have been a plethora of works recently in the deep learning community on layerwise optimizations of different parameters based on their significance to a relevant cost function. For instance, bit widths of weights and activations per layer have been optimized from computation requirement perspective (Garg et al., 2019; Wang et al., 2019; Chakraborty et al., 2020a; Khan et al., 2020; Panda, 2020). Distinct from prior methods, this work explores significance-driven layerwise optimizations for SNN training.

## 3. Preliminaries

### 3.1. Spiking Neural Networks

Let us first consider the algorithmic formulation underpinning ANN-SNN conversion (Rueckauer et al., 2017; Deng and Gu, 2021). In *T* timesteps, for an *N*-layer SNN converted by copying the weights *W*_*n*_ from an ANN (where *n*∈*N*), suppose that a particular neuron in the *n*-th layer at the *t*-th timestep has membrane potential denoted by $V_{n}^{t}$. When the membrane potential is greater than the threshold *Vth*_*n*_, the neuron is reset by subtracting *Vth*_*n*_ from the potential. The membrane potential dynamics of the subtractive IF neurons in response to the input signal $x_{n}^{t}$ for the *n*-th layer can be expressed as the following:


(1) $$\begin{array}{r} V_{n}^{t+1}=V_{n}^{t}+W_{n}*x_{n}^{t}-Vth_{n}*{𝟙}_{V_{n}^{t}>Vth_{n}} \end{array}$$

where, $1_{V_{n}^{t}>Vth_{n}}$ is an indicator function defined as:


(2) $${𝟙}_{V_{n}^{t}>Vth_{n}}\to \{0,1\}=\left\{ \begin{array}{l} 1\text{ if }{\text{V}}_{\text{n}}^{\text{t}}>{\text{Vth}}_{\text{n}}\\ 0\text{ otherwise} \end{array}\right.$$

As the neuron accumulates spikes over time, assuming $V_{n}^{0}=0$, the membrane potential for a particular neuron of the *n*-th layer can be expressed as:


(3) $$\begin{array}{r} V_{n}^{T}=W_{n}*\overset{T}{\underset{t=0}{\sum }}x_{n}^{t}-Vth_{n}*\overset{T}{\underset{t=0}{\sum }}{𝟙}_{V_{n}^{t}>Vth_{n}} \end{array}$$

In the rate encoding framework, the average magnitude of the input spikes over *T* timesteps, ${\hat{x}}_{n}=\overset{T}{\underset{t=0}{\sum }}x_{n}^{t}/T$, represents the equivalent SNN input activation for the *n*-th layer. Simplifying Equation (3),


(4) $$\begin{array}{r} \frac{V_{n}^{T}}{T}=W_{n}*{\hat{x}}_{n}-\frac{Vth_{n}}{T}*\overset{T}{\underset{t=0}{\sum }}{𝟙}_{V_{n}^{t}>Vth_{n}} \end{array}$$

The average input spikes to the (*n*+1)-th layer, ${\hat{x}}_{n+𝟙}$, is the summed indicator function $\overset{T}{\underset{t=0}{\sum }}1_{V_{n}^{t}>Vth_{n}}$. Hence Equation (4) can be rearranged as:


(5) $$\begin{array}{r} {\hat{x}}_{n+1}=\frac{W_{n}*{\hat{x}}_{n}}{Vth_{n}/T}-\frac{V_{n}^{T}}{Vth_{n}} \end{array}$$

Assuming that the remaining $V_{n}^{T}$ will be less than the threshold *Vth*_*n*_ and will not result in a spike, the neuron transfer function can be formulated with a clipping function as the following (Deng and Gu, 2021):


(6) $${\hat{x}}_{n+1}=\frac{1}{T}*clip\left(\left\lfloor \frac{W_{n}*{\hat{x}}_{n}}{Vth_{n}/T}\right\rfloor ,0,T\right)$$

where, a clipping function *clip*(*a, b, c*) restricts the value *a* to be minimally *b* or maximally *c*, and does not affect *a*'s value when *b* ≤ *a* ≤ *c*. As shown in Equation (6), the output of a layer is critically dependent on the threshold *Vth* of the layer and is a bit discretized version of the ReLU functionality, thereby enabling ANN-SNN conversion. It is worth mentioning that this simplification of neuron transfer function may differ slightly from the actual network simulation due to positive and negative membrane potential cancelations (Deng and Gu, 2021) or multiple neuron fan-in (Sengupta et al., 2019).

### 3.2. Differential Evolution Algorithm

Differential Evolution (DE) is a parallel direct search method that optimizes a solution iteratively through evolving candidate solutions. Unlike other optimization techniques such as gradient descent that requires the problem to be differentiable, DE can be applied to noisy and discrete problems. DE starts with a population *P* of initial candidate solutions (randomly initialized or normally distributed around the preliminary solution). In each iteration, the existing candidates are mutated and evaluated by a cost function, and the best ones become members of the next generation. The evolution of new solutions is achieved by two operations, namely “mutation” and “crossover.”

**(i) Mutation** in DE algorithm refers to adding the weighted difference between two candidates to the third. The mutation process to obtain the *i*-th vector ${\vec{v}}_{g+1}$ at generation *g*+1 is given by:


(7) $$\begin{array}{r} {\vec{v}}_{i,g+1}={\vec{x}}_{r1,g}+M\times \left({\vec{x}}_{r2,g}-{\vec{x}}_{r3,g}\right) \end{array}$$

where, ${\vec{x}}_{r1,g}$ is the *r*1-th vector of generation *g*, *r*1, *r*2, *r*3∈{1, 2, .., *P*} are random indices in the population. *M*∈[0, 2] is a real-valued hyper-parameter controlling the extent of mutation in differential variation.

**(ii) Crossover** adds diversity by creating a trial vector ${\vec{u}}_{i,g+1}$ with problem dimension *D* at generation *g*+1:


(8) $$\begin{array}{r} {\vec{u}}_{i,g+1}=\left[u_{i,g+1}\left(1\right),u_{i,g+1}\left(2\right),\ldots ,u_{i,g+1}\left(D\right)\right] \end{array}$$

in which,


(9) $${\text{ u}}_{i,g+1}\left(j\right)\text{ =}\left\{ \begin{array}{l} v_{i,g+1}(j)\text{ if}[rand(j)\leq C]\text{ or }j=randInd({\vec{v}}_{i,g+1})\\ x_{i,g}(j)\text{ otherwise} \end{array}\right.$$

where, *j*∈1, 2, ..., *D*, *u*_*i, g*+1_(*j*) is the *j*-th element of the trial vector ${\vec{u}}_{i,g+1}$, *rand*(*j*) is a real-valued uniform random number generator (RNG) outcome with the range [0, 1] evaluated at *j*-th time; *C* is another real-valued hyper-parameter that controls the extent of inheritance from the mutant vector ${\vec{v}}_{i}$ in the trial vector ${\vec{u}}_{i}$. $randInd\left({\vec{v}}_{i,g+1}\right)$ randomly selects an index from the given vector's dimension 1, 2, ..., *D* and the condition after “or” enforces that there is at least one element from ${\vec{v}}_{i}$. The candidate solution ${\vec{u}}_{i,g+1}$ will be evaluated against ${\vec{x}}_{i,g}$ on the same cost function and the one with the lower cost will be selected as the member of (*g*+1)-th generation. Considering that the DE solution takes *G* generations to converge, the total number of function evaluations (*nfe*) during the optimization process is therefore given by:


(10) $$\begin{array}{r} \text{nfe}=G\times P \end{array}$$

In this work, we used the DE implementation by a Python-based toolkit “SciPy” (Virtanen et al., 2020), which is based on the algorithm outlined in Storn and Price (1997).

## 4. Neuroevolution Guided Hybrid SNN Training Algorithm

As discussed previously, our proposed neuroevolution optimized SNN models are trained using a hybrid approach—(i) standard ANN-SNN conversion (Lu and Sengupta, 2020) followed by (ii) DE optimization of SNN neuron thresholds. The DE optimization is driven by a cost-function evaluated on randomly chosen subsets from the training set. The random shuffling of the sub-dataset adds a regularization effect to the training process. Next, we discuss our cost-function formulation for handling the accuracy-latency tradeoff in standard image classification tasks. We utilize a similar approach for adversarial attack scenarios on the same dataset and show that the thresholds adapt to the new cost-function, thereby showing the flexibility of the approach. Finally, we also provide insights to explain the optimal threshold choice. A detailed implementation of our proposed method is shown in Algorithm 1.

**Algorithm 1:** DE guided hybrid SNN training algorithm.

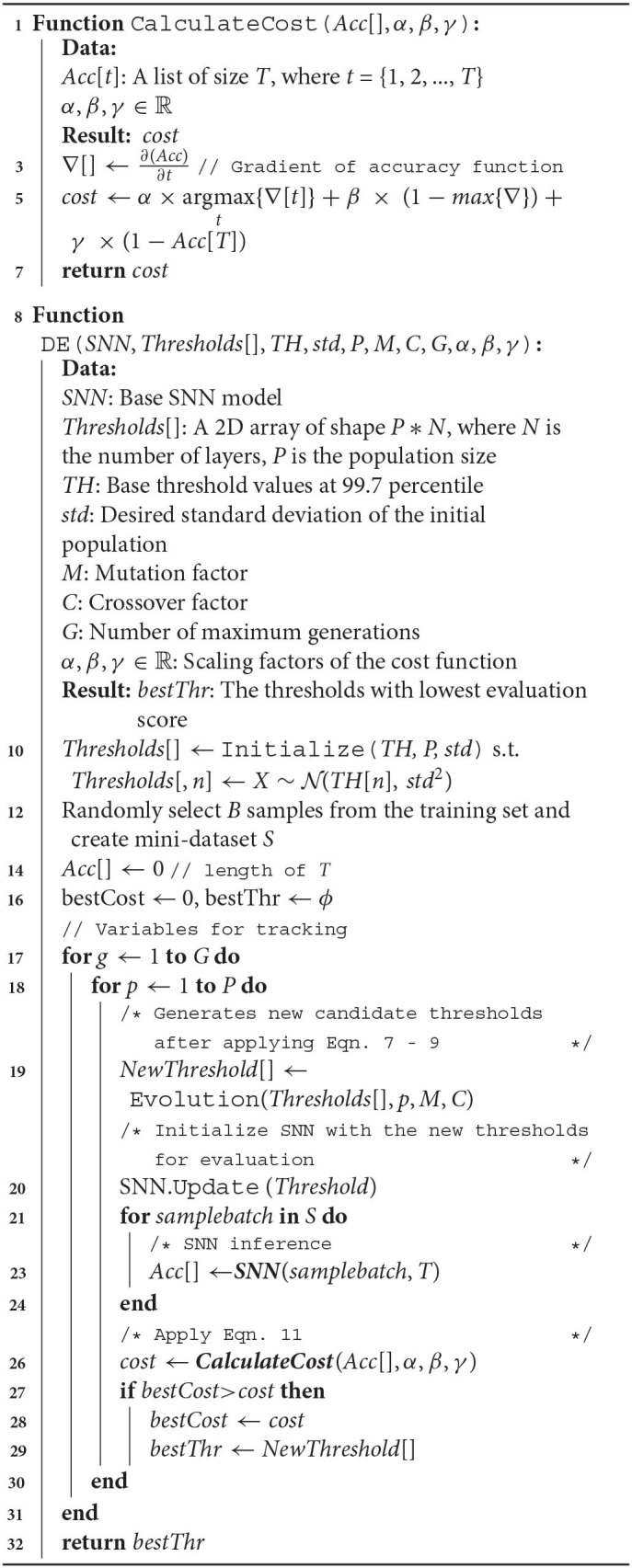

### 4.1. Latency-Accuracy Tradeoff Driven Optimization and Interpretibility

**Figure 1:**
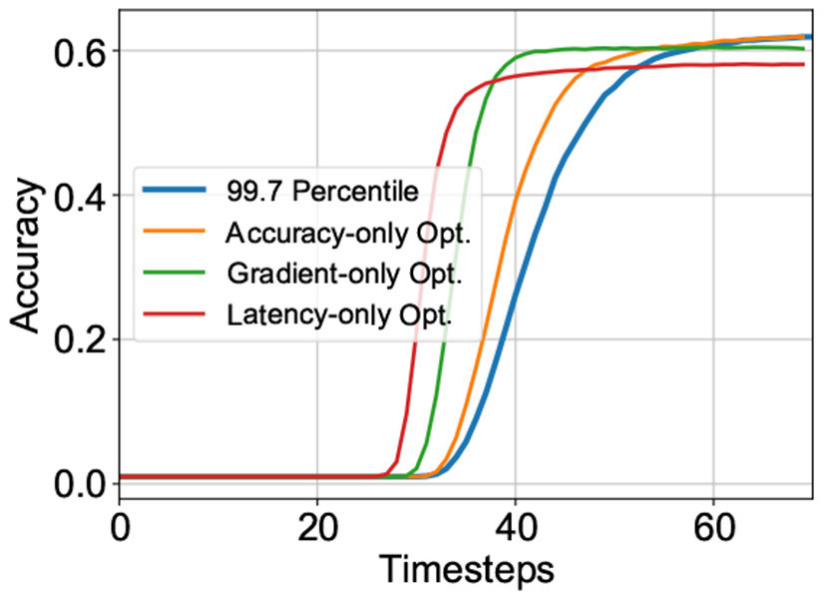
Impact of various components of the cost function on the accuracy-latency tradeoff for VGG-15 model on CIFAR-100 dataset.

Our multi-objective DE cost-function consists of weighted factors to optimize the latency of the SNN along with the final accuracy. In particular, the latency is abstracted by the timestep at which it reaches the highest gradient in the accuracy-time variation function. The resulting costs are scaled by hyperparameters (α, β, and γ) and then linearly summed up. To summarize, the cost function is as follows:


(11) $$\begin{array}{r} Cost=\alpha \times J+\beta \times \left[1-max\left(\nabla \right)\right]+\gamma \times \left(1-Acc\left[T\right]\right) \end{array}$$

where, *J* is argmax_*t*_{∇*Acc*(*t*)}, the timestep at which the SNN reaches the highest gradient in accuracy *max*(∇) with respect to time. The maximal gradient magnitude is also added to the cost function to guide solutions toward models with sharper accuracy-timestep transitions such that latency required to reach a specific accuracy is minimized. We observed that this was critical to achieving the latency-accuracy tradeoff. Finally, the cost function also includes *Acc*[*T*], the final accuracy of the model at timestep *T* (a sufficiently long time window for inference) where *Acc*[] is a function of accuracy against time. It is worth reiterating here that the accuracy is evaluated over randomly chosen subsets of the training set for each candidate solution. The impact of each individual component in the cost function is depicted in Figure 1.

**Figure 2:**
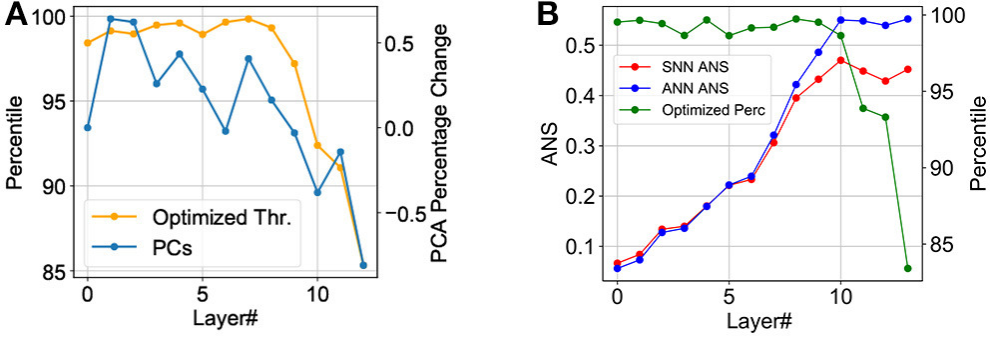
The thresholds are expressed as the percentile of the maximum ANN activations. Both the figures are plotting one of the best solutions in their respective scenarios. **(A)** The optimized threshold shows a similar general trend as the principal components. **(B)** Blue and red: layerwise ANS value (left vertical axis) of the ANN and the converted + optimized SNN, respectively. ANS is significantly reduced after optimization. Green: The optimized threshold (right vertical axis) shows drastic reduction after layer 10 corresponding to the layers where the ANS metric is significantly reduced.

Figure 2A depicts the optimized threshold [expressed as a percentile of the maximum ANN activation (Lu and Sengupta, 2020): higher percentile values translate to higher threshold] as a function of layer number for a typical run. In order to attain an understanding for the importance of layerwise neuron threshold optimization, we hypothesized that this might be correlated to the significance of a particular layer toward its prediction capability. For this purpose, we used Principal Component Analysis (PCA)—one of the prominent tools that can be used to quantify a neural network layer's significance (Chakraborty et al., 2020b). In short, PCA can be thought of as an orthogonal transformation that maps uncorrelated variables in the input data points and forms a basis vector set that maximizes the variance in different directions. Generally, neural network models project the input into higher dimensions as layers get deeper with the goal of achieving linear separability at the final output layer. Therefore, the calculation of the Principal Components (PCs) of each layer's feature map is able to quantify the projective ability of each layer and thus its significance.

We performed PCA on the feature maps before the non-linear activation to examine the redundancy in every layer as the dimension increases. To explain the first, say *R%*, of the variance in the feature map of the layer, only a number of the PCs, denoted by *k*, are needed. Given an activation map ℙ with dimension *B*×*H*×*W*×*F*, where *B* is the mini-batch size, *H* and *W* are height and width of the filter, respectively, and *F* is the number of filters, it is first flattened to 1D in the first three dimensions. This makes the activation ℚ a 2D matrix with dimension *K*×*F* where *K* = *B*×*H*×*W*. Singular value decomposition is applied to ℚ^*T*^ℚ to obtain *L* eigenvectors *v*_*i*_ and eigenvalues λ_*i*_. The total variance *P*_*var*_ is given by:


(12) $$\begin{array}{r} P_{var}=\overset{L}{\underset{i=1}{\sum }}\sigma_{ii}^{2} \end{array}$$

The significance of component λ_*i*_ would be simply $\frac{\lambda_{i}}{P_{var}}$. The first *k* principal components explain variance of a threshold value *R*:


(13) $$\begin{array}{r} R=\frac{\sum_{i=1}^{k}\lambda_{i}^{2}}{\sum_{i=1}^{L}\lambda_{i}^{2}} \end{array}$$

The ratio *R* is used as a threshold for the algorithm to calculate the first *k* PCs and *k* suggests the number of significant components required after removing the redundancy in ℚ. After obtaining *k* PCs for every layer in the SNN to explain a fixed threshold of *R%* variance (99.9% in our case), we interpreted a layer's significance to be proportional to the percentage increase in the number of PCs in comparison to the previous layer, i.e., the layer contributes significantly to the transformation of the input data provided to it by the previous layer. The percentage layerwise changes in PCs are plotted in Figure 2A, and interestingly the general trend matches with the variation of layerwise optimized neuronal thresholds. This is explainable since a higher spiking threshold allows more time for evidence integration, thereby improving SNN accuracy by ensuring more significant layers perform more accurate computations.

### 4.2. Adversarial Attack Driven Optimization and Interpretability

Next, we show that neuron threshold optimization is not application agnostic, thereby requiring the need for a cross-stack optimization. To substantiate our motivation, we consider SNN adversarial attack scenarios. Adversarial attack in neural networks refers to malicious attempts to mislead the model prediction. Since neural networks are proven to be vulnerable in such attacks (Madry et al., 2017), it becomes a non-trivial task to optimize the model for adversarial scenarios. While there are a plethora of adversarial attack algorithms (Chakraborty et al., 2018), we used the vanilla version of the Fast Gradient Sign Method (FGSM) attack as a proof of concept for our optimization method's adversarial robustness. Details in the adversarial setup and implementation will be discussed in the next section.

We applied our neuroevolutionary guided SNN training strategy in this case but optimized for adversarial accuracy-latency tradeoff. Figure 2B depicts the optimized threshold (expressed as a percentile of the maximum ANN activation) as a function of layer number for a typical run. However, the trend shows a slightly different distribution of thresholds as compared to the normal accuracy scenario. We observe that the deeper layers exhibit a similar downward trend of thresholds but this occurs only after layer 10 in the adversarial scenario whereas the network optimized for normal accuracy shows this trend much before (explained by % changes in PCs, as discussed in the previous subsection).

To explain this trend, we used Adversarial Noise Sensitivity (ANS), *A*_δ_, as a metric for measuring layerwise perturbation in neural networks (Panda, 2020). It is defined as the error ratio between a particular layer's perturbed adversarial activation and the unperturbed original activation and can be expressed by the following equation:


(14) $$\begin{array}{r} {𝔸}_{\delta ,n}=\frac{||a_{adv}^{n}-a^{n}|{|}_{2}}{||a^{n}|{|}_{2}} \end{array}$$

where, *a*^*n*^ is the activation map of the *n*-th layer and the subscript *adv* denotes the same activation with adversarial input. In summary, the higher the ANS value of a particular layer, the higher is the sensitivity to noise of that layer. In other words, the layers with high ANS values will perform worse than the layers with low ANS values under the same degree of adversarial attack. In the SNN case, we use the cumulative spikes as the activation:


(15) $$\begin{array}{r} {𝔸}_{\delta ,n}=\frac{||\sum_{t=1}^{T}x_{adv,t}^{n}-\sum_{t=1}^{T}x_{t}^{n}|{|}_{2}}{||\sum_{t=1}^{T}x_{t}^{n}|{|}_{2}} \end{array}$$

where, $x_{t}^{n}$ is the *n*-th layer's spike at timestep *t* and *adv* still denotes the adversarial version; *T* is the total duration of inference. We can observe from Figure 2B that the highest ANS values start from layer 10, which incidentally correlates with the trend of layerwise optimized neural thresholds.

To understand the relationship between neuron threshold and noise sensitivity, one needs to consider the activation discretization caused by the firing threshold. As shown in Equation (6), the output of a layer is critically dependent on the threshold *Vth* of the layer and is a bit discretized version of the ReLU functionality. Thus, the SNN neuron activation representation can be considered to be discretized due to the spiking behavior. When the threshold is lower in the denominator of Equation (6), there will be more discrete states and vice versa. Therefore lower firing threshold should relate to layers with higher noise sensitivity since reduced precision/discretization results in minimizing the adversarial perturbation (Rakin et al., 2018; Sen et al., 2020). To summarize, in adversarial scenarios, the optimal set of thresholds attributes low thresholds to high ANS layers to increase discretization to resist the effect of adversarial noise.

## 5. Experiments and Results

### 5.1. Datasets and Implementation

We evaluated our proposal on the CIFAR-10, CIFAR-100 (Krizhevsky et al., 2009), and the large-scale ImageNet (Deng et al., 2009) dataset. CIFAR-10 and CIFAR-100 datasets consist of 10 and 100 classes, respectively. They include 60,000 32 ×32 colored images partitioned into 50,000 and 10,000 training and testing images respectively. ImageNet 2012 is a much more challenging dataset with 1,000 object categories that include 1.28 million images for training and 50,000 images for validation. The ImageNet images are randomly cropped into 224 ×224 pixels before being fed into the network. All images are normalized with zero mean and unit variance and shuffled during the training and DE optimization phase. The ANN models are pretrained VGG15 architectures based on constraints described in our prior work (Lu and Sengupta, 2020). All experiments are conducted in “PyTorch” framework using “BindsNet” toolbox (Hazan et al., 2018) with the “SciPy” toolbox providing efficient DE algorithm implementation.

For the adversarial attack scenario, we used FGSM as a white box attack where the model parameters and network structure are fully available to the attacker. It utilizes the gradient of the original input and then perturbs it to create an adversarial version that maximizes the loss. This perturbation process can be summarized as:


(16) $$\begin{array}{r} \hat{X}=X+\epsilon \times sign\left[\nabla_{X}L\left(w,X,y\right)\right] \end{array}$$

where, $\hat{X}$ is the perturbed image, *X* is the original input image, ϵ is the hyper-parameter to adjust the extent of perturbation, ∇_*X*_*L*(*w, X, y*) is the gradient of the loss *L* given model parameter *w*, input *X* and label *y*, *sign*() operation provides the direction of the gradient (in terms of “1”s and “−1”s). In our case, we adopted ϵ = 8/255, commonly used in other works. Further details can be found in Goodfellow et al. (2015).

### 5.2. Results

The specific hyperparameter settings for our algorithm are specified in Table 1 for the various datasets and applications. For the DE algorithm, we used a typical setting of the mutation rate (*M* is a random number between 0.5 and 1.5) and crossover rate (*C* = 0.7). It is worth reiterating here that only the training set is utilized during the neuroevolutionary optimization process. Considering that the DE algorithm is initialized with *P* particles and takes *G* generations to converge (measured by averaging over 20 runs), the excess overhead of running our hybrid training technique is tabulated as “Training Cost” in Table 1 and is computed in terms of the training set size by:


(17) $$\begin{array}{r} Training\;Cost=\left(E\times G\times P\right)/D_{train} \end{array}$$

where, *E* is the total number of images used for cost function evaluation per particle per generation and *D*_*train*_ is the total number of images in the training set. Table 1 illustrates the advantage of our proposed algorithm in terms of scalability. The number of evaluation images required for the optimization process is primarily determined by the dimensionality of the optimization space rather than the size of the training set of the dataset. Hence, the “Training Cost” reduces significantly for complex datasets like ImageNet. This is in stark contrast to BPTT guided hybrid training approaches where backpropagation based gradient updates will require significantly large training datasets.

**Table 1:** Algorithm hyperparameters for various datasets.

**Dataset**	**α**	**β**	**γ**	**Initial Perc**.	***std***	**Population Size *P***	**No. of Generations *G***	***nfe***	Training Cost^*^
**Image classification**									
CIFAR-10	1	10	500	99.7	0.15	25	25.9	647.5	38.85
CIFAR-100(VGG15)	1	40	20	99.7	0.15	25	26.55	663.75	39.825
CIFAR-100(VGG11)	0.7	60	200	99.7	0.13	35	7.8	312	16.38
ImageNet	1	70	110	99.8	0.13	20	6.08	121.66	0.475
**Image classification with adversarial attack**									
CIFAR-10	1	2	60	99.7	0.25	25	21.94	548.5	13.2
CIFAR-100	1	2	60	99.7	0.25	25	24.22	605.5	14.5

* *Training cost computed using Eqn. 17*.

**Figure 3:**
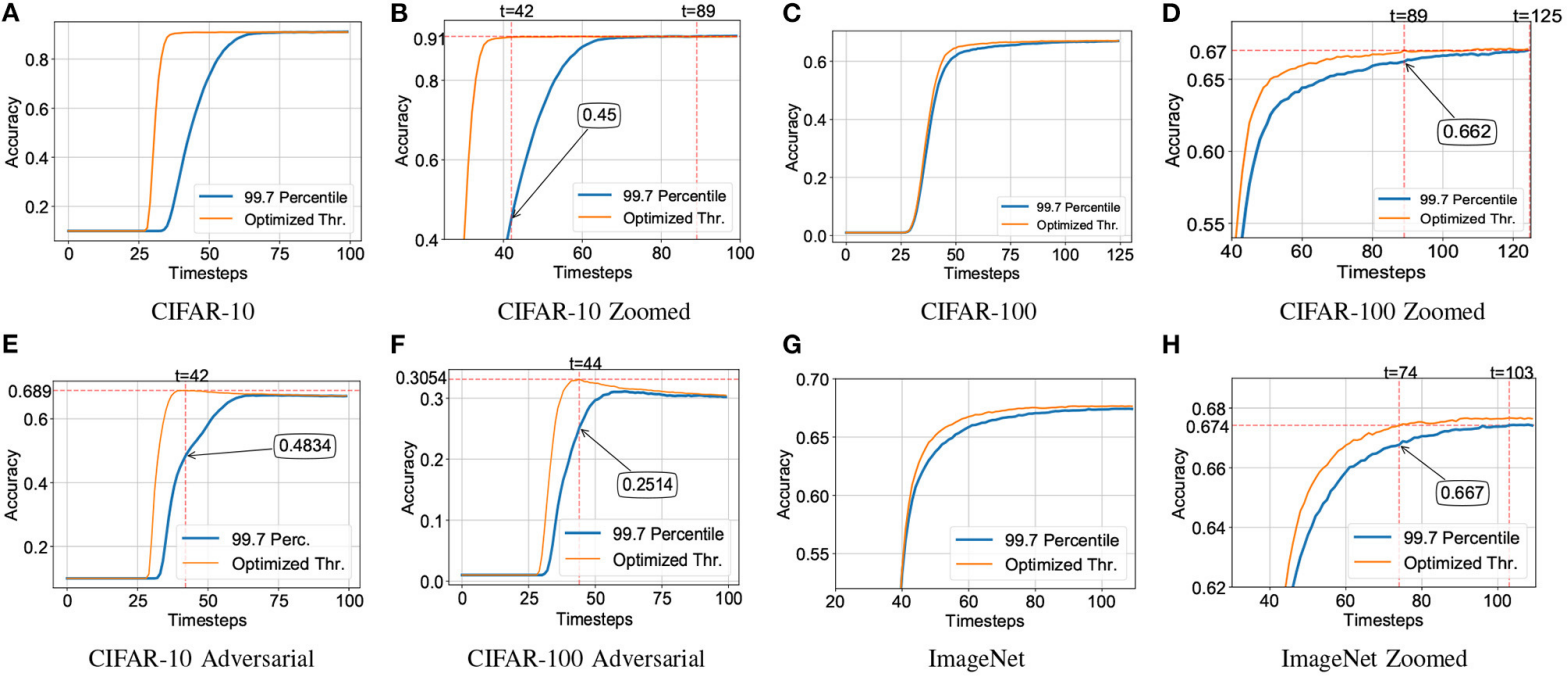
Accuracy vs. timesteps for neuroevolutionary optimized SNN against homogeneously normalized (using 99.7 percentile of maximum activation; Lu and Sengupta, 2020) SNN on CIFAR-10 dataset. Iso-time and iso-accuracy comparison are denoted by dotted-red line and textboxes. **(A)** CIFAR-10, **(B)** CIFAR-10 Zoomed, **(C)** CIFAR-100, **(D)** CIFAR-100 Zoomed, **(E)** CIFAR-10 Adversarial, **(F)** CIFAR-100 Adversarial, **(G)** ImageNet, **(H)** ImageNet Zoomed.

The performance of our proposed hybrid SNN training technique for CIFAR-10, CIFAR-100, and ImageNet datasets are depicted in Figure 3 including adversarial attack scenarios. Significant latency improvement is consistently observed in all cases in contrast to a uniform percentile-based threshold optimization scheme. Iso-accuracy and iso-latency improvements for latency and accuracy, respectively are also provided. A detailed comparison of the performance of our algorithm against prior work is provided in Tables 2, 3.

**Table 2:** Performance benchmarking of our proposal against prior works.

**References**	**Method**	**Architecture**	**SNN accuracy (%)**	**Timesteps**
**CIFAR10**				
Hunsberger and Eliasmith (2015)	ANN-SNN	2C, 2L	82.95	6, 000
Sengupta et al. (2019)	ANN-SNN	VGG16	91.55	2, 500
Kim et al. (2018)	Phase-coding	VGG16	91.2	1, 500
Park et al. (2019)	Burst-coding	VGG16	91.4	1, 125
Park et al. (2020)	Time-Till-First-Spike	VGG16	91.40	680
Rueckauer et al. (2017)	ANN-SNN	4 Conv, 2 FC	90.85	400
Cao et al. (2015)	ANN-SNN	3C,2L	77.43	400
Rathi et al. (2020)	Hybrid	VGG16	92.02	200
Lee et al. (2020)	Backprop	VGG9	90.45	100
Lu and Sengupta (2020)	ANN-SNN	VGG15	91.03	91
This work	Neuroevolutionary SNNs	VGG15	91.05	42
**CIFAR100**				
Kim et al. (2018)	Phase-coding	VGG16	68.6	8, 950
Park et al. (2019)	Burst-coding	VGG16	68.77	3, 100
Han et al. (2020)	ANN-SNN	VGG16	70.09	768
Park et al. (2020)	Time-Till-First-Spike	VGG16	68.8	680
Rathi et al. (2020)	Hybrid	VGG11	67.87	125
Lu and Sengupta (2020)	ANN-SNN	VGG11	67.00	125
This work	Neuroevolutionary SNNs	VGG11	67.00	89
**ImageNet**				
Sengupta et al. (2019)	ANN-SNN	VGG16	69.96	2, 500
Han et al. (2020)	ANN-SNN	VGG16	71.34	768
Rueckauer et al. (2017)	ANN-SNN	VGG16	49.61	400
Rathi et al. (2020)	Hybrid	VGG16	65.19	250
Lu and Sengupta (2020)	ANN-SNN	VGG15	67.40	103
This work	Neuroevolutionary SNNs	VGG15	67.40	74

**Table 3:** Performance benchmarking of our proposal against prior works for SNN adversarial attacks. All FGSM are white-box attacks and use ϵ = 8/255.

**References**	**Attack**	**Method**	**Architecture**	**ANN (%)**	**SNN (%)**	**Timesteps**
**CIFAR10**						
Sharmin et al. (2020)	FGSM	Backprop	ResNet20	1.8	3.8	200
Sharmin et al. (2020)	FGSM	Backprop	VGG5	10.4	15.0	100
Sharmin et al. (2019)	FGSM	Backprop	VGG9	61.7	51.6	70
Lu and Sengupta (2020)	FGSM	ANN-SNN	VGG15	67.42	67.40	74
This work	FGSM	Neuroevolutionary SNNs	VGG15	67.42	68.9	42
**CIFAR100**						
Sharmin et al. (2020)	FGSM	Backprop	VGG11	17.1	15.5	200
Lu and Sengupta (2020)	FGSM	ANN-SNN	VGG15	30.54	31.11	61
This work	FGSM	Neuroevolutionary SNNs	VGG15	30.54	33.1	44

### 5.3. Comparison Against Backpropagation Through Time (BPTT) Fine-Tuning

**Figure 4:**
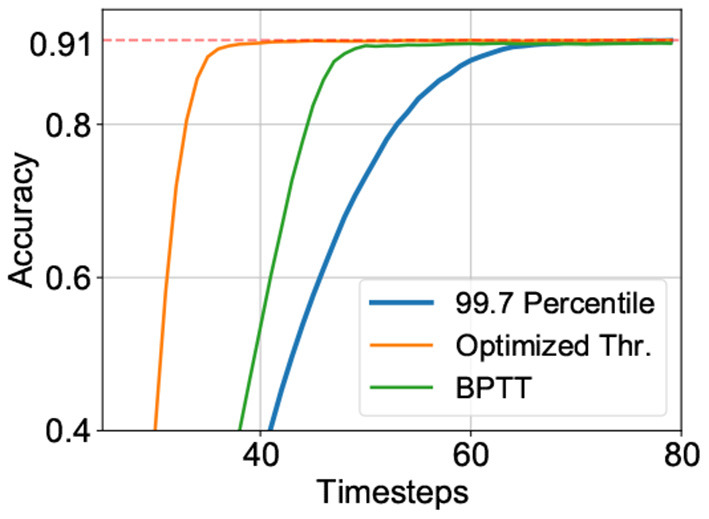
Performance of VGG15 model on the CIFAR-10 dataset based on various training techniques—(blue) ANN-SNN conversion: 99.7 Percentile, (orange) Hybrid neuroevolutionary approach: Optimized Thr., and (green) BPTT: Hybrid training with backpropagation through time.

As mentioned before, our work is most relevant to hybrid SNN training approaches where the network is fine-tuned using BPTT after conversion (Rathi and Roy, 2020; Rathi et al., 2020). While the computational overhead is significantly higher in BPTT based approaches, another important difference between the two approaches lies in the absence of any temporal information in our neuroevolutionary optimization process. In order to benchmark the performance of the two hybrid training techniques, we performed BPTT fine-tuning from the same initialized converted SNN model as used in our neuroevolutionary algorithm. For BPTT, the network layers are unfolded at each timestep for IF operations. The BPTT method uses surrogate gradient for IF neurons (Bellec et al., 2018):


(18) $$\begin{array}{r} \frac{\partial p_{i}^{t}}{\partial V_{i}^{t}}=\gamma max\left\{0,1-|V_{i}\left(t\right)|\right\} \end{array}$$

where, *p* is the output spike train, *V*_*i*_(*t*) is the normalized membrane potential voltage of neuron *i* at timestep *t*. γ is a hyper-parameter to dampen the error which is set to 0.15 in our case. For our experiments, we used a pre-trained VGG15 model on CIFAR-10 dataset, initialized with 99.7 percentile thresholds for the IF neuron layers. The BPTT algorithm was run for 25 epochs and the network was unrolled over 70 timesteps. However, as shown in Figure 4, while the hybrid BPTT training performed better than a simple conversion approach, it was outperformed by our proposed hybrid neuroevolutionary approach. While recent versions of hybrid BPTT training (Rathi and Roy, 2020) have reported only 5 timesteps as SNN latency, it is probably attributed to performing gradient descent on additional introduced parameters like neuron leak. Further, latency is re-defined to exclude intrinsic delay of an SNN where the neuron in each layer spikes at the current timestep instead of the next, and therefore eliminates the intrinsic layerwise SNN delay. While this is a simple method to reduce SNN latency, it may potentially have limitations in neuromorphic chip designs in terms of spike routing or parallel spike processing capability. It is worth mentioning here that additional optimizations like learnable membrane time constants (Rathi and Roy, 2020; Fang et al., 2021b), network architectures like Residual networks (Fang et al., 2021a), conversion error calibration techniques (Deng and Gu, 2021; Li et al., 2021), hybrid spike encoding (Datta et al., 2021) are complementary to the current proposal and can be augmented in the algorithm to further minimize the inference latency. Tables 2, 3 therefore includes primarily basic SNN architectures based on IF nodes without any additional optimizations to substantiate the importance and interpretability of the need for layerwise threshold optimization. The dimensionality of the optimization algorithm can be easily expanded to incorporate additional optimization parameters like membrane potential leak, spike encoding rate, among others.

**Figure 5:**
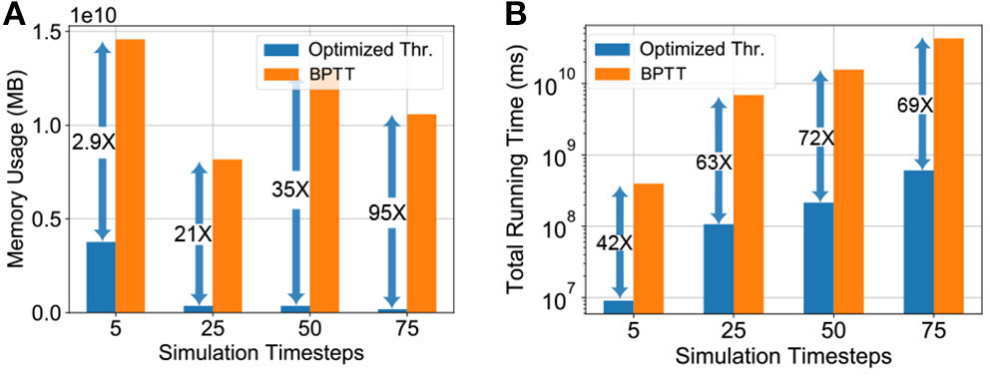
Memory usage and running time comparison of hybrid neuroevolutionary and BPTT based approaches of VGG-15 model on ImageNet dataset, with maximum batch-size (21, 2, 2, 1) for (5, 25, 50, 75) timesteps, respectively. **(A)** Memory usage, **(B)** Expected running time.

To quantitatively substantiate the computational benefit of our proposed hybrid neuroevolutionary training approach against BPTT based methods, we also report the memory usage and running time of the two methodologies on the ImageNet dataset in Figure 5. The memory usage was profiled and the extrapolated running time for our proposed neuroevolutionary algorithm is calculated as:


(19) $$\begin{array}{r} Total\;Running\;Time=\bar{\tau }\times Training\;Cost\times \frac{D_{train}}{B} \end{array}$$

where, $\bar{\tau }$ is the average running time per batch (averaged over 20 batches), “Training Cost” is calculated from Equation (17) and *B* is the batch-size. The average running time $\bar{\tau }$ is used to minimize the fluctuations caused by external processes. The “Training Cost” of BPTT was considered to be 20 epochs as reported in prior literature (Rathi et al., 2020). It is worth mentioning here that unlike our proposed algorithm, BPTT is heavily memory-constrained for large scale datasets like ImageNet even for 5 timesteps, as shown in Figure 5. Our algorithms were run on Nvidia Tesla V100 16 GB GPUs where we had to limit the batch-size for the BPTT based hybrid training approach due to memory limit. The batch-sizes of Figure 5 are chosen based on the maximum memory capacity of the BPTT based approach and iso-batch-size comparison is performed with the neuroevolutionary method. As illustrated in the plot, the situation worsens significantly with increasing timesteps due to drastic increase in gradient trace information. As shown in Figure 5, our proposed neuroevolutionary method requires 2.9 × less memory and 42 × less running time than BPTT based framework even for five timesteps used for SNN simulation. It is worth mentioning here that iso-timestep based comparison may not be valid for further optimized SNN algorithms like BPTT with membrane potential leak (Rathi and Roy, 2020; Fang et al., 2021b) and therefore require further benchmarking.

## 6. Conclusions

In conclusion, the work explores a neuroevolution-based hybrid SNN training strategy that optimizes SNN specific parameters like neuron spiking threshold after the conversion process. While significantly outperforming state-of-the-art approaches in terms of accuracy-latency tradeoffs in image classification tasks including adversarial attack scenarios, the work highlights the need for significance-driven layerwise SNN optimization schemes leading to explainable SNNs. We also highlight that the work outperforms computationally expensive BPTT based fine-tuning approaches since temporal information may not be relevant in static image classification tasks. Future exploration into application drivers with temporal information (Mahapatra et al., 2020; Singh et al., 2021) or temporal spike encoding schemes (Petro et al., 2020; Yang and Sengupta, 2020) is expected to truly leverage the full potential of BPTT based SNN training strategies.

## Data Availability Statement

The raw data supporting the conclusions of this article will be made available by the authors, without undue reservation.

## Author Contributions

AS developed the main concepts. SL performed all the simulations. All authors assisted in the writing of the paper and developing the concepts.

## Funding

This work was supported in part by the National Science Foundation grants CCF #1955815, BCS #2031632, and ECCS #2028213 and by Oracle Cloud credits and related resources provided by the Oracle for Research program.

## Conflict of Interest

The authors declare that the research was conducted in the absence of any commercial or financial relationships that could be construed as a potential conflict of interest.

## Publisher's Note

All claims expressed in this article are solely those of the authors and do not necessarily represent those of their affiliated organizations, or those of the publisher, the editors and the reviewers. Any product that may be evaluated in this article, or claim that may be made by its manufacturer, is not guaranteed or endorsed by the publisher.
